# Neurofeedback: new territories and neurocognitive mechanisms of endogenous neuromodulation

**DOI:** 10.1098/rstb.2023.0081

**Published:** 2024-10-21

**Authors:** James Sulzer, T. Dorina Papageorgiou, Rainer Goebel, Talma Hendler

**Affiliations:** ^1^ Department of Physical Medicine and Rehabilitation, MetroHealth Medical Center and Case Western Reserve University, Cleveland, OH, USA; ^2^ Departments of Psychiatry, Neuroscience, Physical Medicine & Rehabilitation, Dan L. Duncan Comprehensive Cancer Center, Center for Space Medicine, Baylor College of Medicine, Houston, TX, USA; ^3^ Department of Electrical and Computer Engineering, Neuroengineering Research Initiative, Rice University, Houston, TX, USA; ^4^ Department of Cognitive Neuroscience, Faculty of Psychology and Neuroscience, Maastricht University, Maastricht, The Netherlands; ^5^ School of Psychological Science, Faculty of Health and Medical Science and Sagol School of Neuroscience, Tel Aviv University, Tel Aviv, Israel; ^6^ Sagol Brain Institute, Tel Aviv Sourasky Medical Center, Tel Aviv, Israel

**Keywords:** operant conditioning, neurofeedback, neuroimaging, fMRI, EEG, fNIRS

## Abstract

Neurofeedback (NF) is endogenous neuromodulation of circumscribed brain circuitry. While its use of real-time brain activity in a closed-loop system is similar to brain–computer interfaces, instead of controlling an external device like the latter, the goal of NF is to change a targeted brain function. In this special issue on NF, we present current and future methods for extracting and manipulating neural function, how these methods may reveal new insights about brain function, applications, and rarely discussed ethical considerations of guiding and interpreting the brain activity of others. Together, the articles in this issue outline the possibilities of NF use and impact in the real world, poising to influence the development of more effective and personalized NF protocols, improving the understanding of underlying psychological and neurological mechanisms and enhancing treatment precision for various neurological and psychiatric conditions.

This article is part of the theme issue ‘Neurofeedback: new territories and neurocognitive mechanisms of endogenous neuromodulation’.

## 1. Introduction

Neurofeedback (NF) is endogenous neuromodulation of circumscribed brain circuitry. While its use of real-time brain activity in a closed-loop system is similar to brain–computer interfaces, instead of controlling an external device like the latter, the goal of NF is to change a targeted brain function. NF is a conditioning procedure that requires reinforcement learning [1], most often driven by a visual representation of the modulated brain function. The concept of voluntary control over neural function has been investigated since the 1950s [2] using electroencephalography (EEG). In subsequent decades, recording modalities expanded to single neuron recordings in animals [3], multi-electrode arrays [4], functional magnetic resonance imaging (fMRI) [5], functional near-infrared spectroscopy (fNIRS) [6] and magnetoencephalography (MEG) [7]. Computational power and real-time processing capabilities have advanced in parallel. This dramatic growth in capabilities combined with our increasing awareness of the high prevalence and limited therapeutic solutions of neurological and psychological issues in our society has generated a swell of excitement over non-pharmacological, yet brain-driven and precise treatments [8–10]. Where has this excitement taken us? In this special issue on NF, we present current and future methods for extracting and manipulating neural function, how these methods may reveal new insights about brain function, applications and rarely discussed ethical considerations of guiding and interpreting the brain activity of others. Together, the articles in this issue outline the possibilities of NF use and impact in the real world, poising to influence the development of more effective and personalized NF protocols, improving the understanding of underlying psychological and neurological mechanisms and enhancing treatment precision for various neurological and psychiatric conditions.

## 2. Methodological advancements

Some of the most exciting advancements in the field of NF come from new methodological approaches. This includes improvement in core elements in reinforced self-neuromodulation practices such as the precision of neural targeting or the contextual representation of multi-dimensional information of the modulated brain function.

In the article entitled ‘Closed-loop fMRI at the mesoscopic scale of columns and layers: can we do it and why would we want to?’, Chaimow *et al*. offer a future perspective on the potential benefits of using 7 T fMRI for probing a neural target [11]. One of the primary advantages of fMRI is its relatively high spatial resolution compared to other non-invasive imaging techniques. With sub-millimetre resolution, it becomes possible to functionally differentiate cortical layers and cortical columns regarded as the brain mesoscopic scale. Can NF at high-field MRI (7 T and higher) be used to non-invasively examine the neurophysiology at the mesoscopic scale? For example, the concept of NF targeting cortical laminar level may be able to influence mental health disturbances originating in different cortical layers (e.g. schizophrenia). Further, NF enables brain activity to be used as an independent variable, enabling a better understanding of the functional role of cortical layers. However, there are significant practical hurdles to overcome before realizing mesoscopic NF, including noise, specificity and data processing. The authors discuss these challenges in detail and offer a vision for how to pursue this exciting direction.

Evidence about induced neuromodulation by NF points to its potential for driving neuroplasticity in humans. In the article titled ‘Psilocybin-assisted neurofeedback for the improvement of executive functions: a randomised semi-naturalistic-lab feasibility study’, Enriquez-Geppert *et al.* examined the effects of psilocybin, a serotonergic psychedelic drug, on EEG-NF learning and outcome [12]. Psilocybin has recently gained traction in the neuroscience community for its effects on neuroplasticity. In a placebo-controlled randomized trial, the authors examined whether the application of the psychedelic drug before one session of NF affects learning trajectories including retention and the behavioural outcome of executive function. The combination of neuropharmacology and NF could be used in the future to extend a window of increased neuroplasticity and through that could improve psychiatric conditions and perhaps beyond.

The manuscript titled ‘Individualized fMRI neuromodulation enhances visuospatial perception: a proof-of-principle study’ by Papageorgiou and colleagues investigates the effectiveness of individualized fMRI neuromodulation (iNM) in enhancing visuospatial perception by targeting visual imagery, motor planning and selective exteroceptive–interoceptive attention [13]. The study involved healthy participants performing a direction discrimination task at supra- and subthreshold levels under iNM and control conditions. This approach targets specific brain regions, with the goal of leveraging the brain’s functional redundancy to enhance neural plasticity and improve visuospatial perception. To assess the causal effects of iNM mechanisms, this study employed encoding and decoding analyses, demonstrating that iNM increased both the area under the curve of the BOLD intensity and classification accuracy for motion direction and coherence across visual perception, visual imagery, motor planning and selective attention networks. The innovation of this study lies in its ability to precisely tailor neuromodulation to each participant’s unique brain anatomy and functional organization, offering a more targeted and efficient method than traditional, generalized approaches. iNM holds significant potential for advancing personalized neuromodulation treatment, which could improve the quality of life for patients with neurological disturbances such as cortical blindness and early cognitive impairment.

In a feasibility study entitled ‘Modulating subjective pain perception with decoded MNI space neurofeedback: a proof-of-concept study’, Berman *et al*. investigated the effectiveness of fMRI NF in modulating pain-related brain activity and influencing subjective pain experiences [14]. The researchers undertook some data processing innovations for multivariate decoding of the whole brain in real-time, over 200 000 voxels. The targeted decoder was correlated to participant-specific subjective ratings of pain. The researchers then trained participants in the upregulation or downregulation of these brain patterns without providing explicit instructions on how to manipulate the feedback signal. Results showed that pain ratings can be predicted in real-time based on brain activity and that the subjective experience of pain can be down-regulated independently from the perception of stimulus intensity.

In the future perspective article entitled ‘Semantic fMRI neurofeedback of emotions: from basic principles to clinical applications’, Goebel *et al.* review the value of applying semantic-based NF to emotion-regulation training [15]. As opposed to mean activity feedback often visualized in a thermometer, semantic NF shows the current mental state of a participant as a point in a two-dimensional map reflecting its distance to pre-measured emotional mental states (e.g. ‘happy’, ‘content’, ‘sad’, ‘angry’). From emotion-inducing localizer runs, the locations of emotions in the semantic map are computed using representational similarity analysis (RSA) of multivariate activity patterns extracted from emotion-related brain areas. During subsequent NF runs, the location of the current mental state is also calculated using RSA and projected in the static semantic emotion map. Participants can then learn to navigate the emotional semantic space by ‘moving’ the point reflecting the current mental state closer to the location of a desired emotional state (e.g. ‘happy’). This innovative approach provides unprecedented transparency of the mental state during self-regulation training and might lead to a new therapy for patients with mood disorders.

In the review entitled ‘From lab to life: challenges and perspectives of fNIRS for hemodynamic-based neurofeedback in real-world environments’, Klein *et al*. delve into the specific advantages and disadvantages of fNIRS NF, particularly in comparison to fMRI NF, and its use in imaging motor and prefrontal cortices, as well as language and social networks [16]. fNIRS NF has been evolving in parallel with fMRI NF, showing promise as a wearable, lower-cost modality of hemodynamic brain imaging and NF that could enable scalable implementation. Practical considerations such as physiological noise, cap placement and probe design are discussed in the context of improving the signal quality. The authors cover applications including ‘hyper-NF’ that connects multiple individuals’ brains or ‘fingerprinting’ that uses fNIRS correlates of higher resolution fMRI activity to indirectly access deep brain areas.

## 3. Mechanisms

How NF works is as yet an open question. It is well established that while an individual is attempting to modulate one’s own targeted brain function, additional regions in the brain are co-modulated. The involvement of such regions in reward, cognitive control and learning processes has led to the suggestion that they serve as a *general domain* in NF. It is as yet unknown how these regions interact with successful target modulation, what is the learning framework for change in modulation capacity and to what degree individual difference contributes to such capacity. Several articles in this issue address these questions by demonstrating results from experiments that examine either a specific mechanism of action, such as neural integration and segregation, or the involvement of accompanied mechanism, such as co-modulated network, associated mental operation and learning.

The paper ‘Mechanisms of brain self-regulation: psychological factors, mechanistic models and neural substrates’ by Sitaram *et al*. reviews the current understanding of brain self-regulation mechanisms through NF and brain–computer interfaces [17]. It addresses the variability in NF learning outcomes and emphasizes the need for better comprehension of cognitive and psychological factors, theoretical models and neural substrates involved in self-regulation. The paper highlights the importance of reinforcement learning and active inference models in understanding self-regulation and calls for more sophisticated and controlled studies to optimize NF protocols and improve clinical and scientific outcomes. Advances in artificial intelligence and machine learning will further refine these techniques, making them more accessible and efficient. The integration of embodied cognition theories may also lead to novel NF approaches that consider brain–body–environment interactions, potentially revolutionizing the field of neurorehabilitation and cognitive enhancement.

The manuscript titled ‘Amygdala self-neuromodulation capacity as a window for process-related network recruitment’ by Gurevitch *et al*. examines the potential of NF targeting downregulation of the amygdala to enhance self-modulation of ‘beyond-the-target’ brain function and through that further improve emotion-regulation process [18]. The study specifically investigates the establishment of neuromodulation capacity during repeated NF sessions targeting the Amygdala Electrical Finger-Print (Amyg-EFP), an fMRI-derived EEG model of amygdala activation. Based on three datasets (total *n* = 97, psychiatric patients and healthy first responders), the results indicate that Amyg-EFP NF improves neuromodulation capacity (i.e. success in downregulating the target), which correlates with post-training downregulation of amygdala fMRI and lower alexithymia (a measure of stress dysregulation). Individual differences in Amygd-EFP neuromodulation capacity were linked to pre-training amygdala fMRI reactivity, initial EFP neuromodulation success, as well as co-modulation in ‘beyond-the-target’ brain regions such as the posterior insula and parahippocampal gyrus, known to be involved in interoception and learning. This study provides insights into the mechanisms of successful self-neuromodulation of the amygdala for emotion regulation, highlighting the importance of individual differences in brain function prior to and due to the training in beyond-the-target regions.

The manuscript ‘Online self-evaluation of fMRI-based neurofeedback performance’ by Muñoz-Moldes *et al*. investigates the subjective evaluation of self-regulation performance using fMRI NF [19]. The study involved participants performing a motor imagery task to modulate the supplementary motor area activity while making performance predictions and confidence judgments. Over three sessions, participants showed improved performance predictions, though confidence levels did not correlate with prediction accuracy. This study provides insights into the cognitive processes underlying self-evaluation in NF, specifically the differentiation between performance predictions and confidence. This research could lead to the development of more effective NF training programmes by incorporating strategies to enhance self-evaluation accuracy. Additionally, this work can guide further research on the metacognitive aspects of NF and brain–computer interfaces, promoting advances in personalized neurotherapy.

The manuscript ‘Inducing representational change in the hippocampus through real-time neurofeedback’ by Peng *et al*. investigates how real-time fMRI NF can be used to induce coactivation between different object representations in the visual cortex and examines the resulting representational changes in the hippocampus and their reflected behavioural manifestations [20]. Participants were trained to activate the representation of one object while viewing another, possibly leading to memory-related representational overlap and difficulty in differentiation. Indeed, the effect of NF on integration was evident in behavioural changes and neural activity patterns in the hippocampus, particularly the CA1 subfield. This study demonstrates that real-time fMRI NF can effectively induce memory modifications by coactivating representations in the visual cortex, leading to significant changes in hippocampal activity. This research points to the potential of NF applications, leading to new treatments for memory-related disorders as well as in education and training programmes.

## 4. Applications

The concept of endogenous neuromodulation over observable brain activity via operant conditioning has understandably produced excitement in the neurological and psychiatric communities. The clinical applications promised by NF are numerous, with use towards theoretically any cognitive or neurological deficit. However, the excitement has outpaced the evidence, in part because of small samples and suboptimal trial designs [21,22]. More recently, applications of NF have been tested in randomized controlled trials (RCTs), including depression [23], tinnitus [24], brain injury rehabilitation [25], post-traumatic stress disorder [26], obsessive compulsive disorder [27], pain [28] and others. While these studies targeted the function of a circumscribed brain region, other efforts have included larger areas and networks, even the whole brain, followed by feature-specific pattern analysis to obtain individual-specific targeted process [29]. This special issue presents examples of machine learning-based NF now translated towards clinical applications in pain, addiction and gaming disorders. Using brain decoders, these approaches help to more accurately target neural mechanisms of brain disorders, but interestingly, they also help as a tool to determine effective causal relationships with underlying neural circuitry.

In the article titled ‘Regulation of craving for real-time fMRI neurofeedback based on individual classification’, Kim *et al*. show the usage of machine learning classifiers of the whole brain with fMRI NF, applied to smoking craving [30]. The study involved 31 participants with smoking use disorder undergoing NF sessions to manage cravings, comparing individual-level versus group-level classifiers. They found that individual-level classifiers provided more accurate and effective NF than group-level classifiers. The findings highlighted the increased accuracy in distinguishing between craving and non-craving states, suggesting the potential of individualized NF in self-regulating smoking cravings and demonstrating the benefits of personalized NF approaches in treating addictive behaviours by targeting specific neural mechanisms.

In the feasibility study titled ‘Decoding and modifying dynamic attentional bias in gaming disorder’ by Oka *et al*., multivariate fMRI NF was used to treat gaming disorder by modifying attentional biases toward gaming stimuli [31]. Researchers combined neuroimaging with an approach-avoidance task customized to individual gaming preferences and used multivariate pattern analysis to identify the insula as a key region involved in attentional bias in a proof-of-concept design. This study showed that training could reduce the attractiveness of gaming stimuli by providing NF based on real-time decoding of insula activity. This study highlights the potential of fMRI NF as a personalized, non-pharmacological treatment and suggests that this method could be applied to other behavioural addictions and attention-related disorders.

One of the most studied applications of NF is attention-deficit/hyperactivity disorder (ADHD), for which NF has a long history with mixed results, particularly in the EEG domain. In the study entitled ‘Effects of fMRI neurofeedback of right inferior frontal cortex (rIFC) on inhibitory brain activation in children with ADHD’, Lukito *et al*. conducted a double-blinded RCT to examine the efficacy of rIFC-fMRI NF in treating ADHD in a sample of 88 boys between 10 and 18 years old focusing on brain activation and inhibitory control [32]. The study compared active and sham fMRI NF for changing brain activation during a well-established inhibitory control task (Stop Task). Results showed no significant differences in rIFC activation between groups or behavioural correlates related to ADHD of brain activation following training. These negative results show the importance of recruiting large and variable samples (i.e. including both genders and different age groups) and testing for process-related outcomes in a robust trial design, which includes sham and/or active controls, calling for careful consideration of the clinical meaning of NF for specific clinical population.

## 5. Ethics and regulatory considerations

The ‘pacing problem’ is the concept that technology will always lead to regulation. Today, we see examples of the pacing problem with autonomous driving, social media and generative artificial intelligence. The two articles on the ethics of NF are examples of trying to get ahead of the pacing problem.

In October 2021, Chile became the first country to recognize neurorights in its constitution. With the ability to guide an individual’s neural activity via NF, there is potential for harm. Ruiz *et al*. discuss key questions in their perspective entitled ‘Neurorights in the Constitution: from neurotechnology to ethics and politics’ [33]. Are the rights to our brain activity moral, legal or both? Are neurorights different from human rights? How do NF and brain–computer interfaces fit within regulatory bounds? What happens when theoretical considerations conflict with practical ones? How do we balance privacy and safety with the potential for medical efficacy?

Which regulatory frameworks should be initiated are discussed by Furnari *et al*. in their article entitled ‘Neurofeedback: potential for abuse and regulatory frameworks in the United States’ [34]. Operant conditioning principles show that neural activity can be manipulated without the individual’s knowledge, offering a potentially harmful scenario and raising the following questions: Are disclaimers sufficient to prevent harm? How much influence over brain activity should be allowed and how is that influence changed by experimental parameters? How do we jump ahead of the pacing problem? Should we react with laws based on a threshold or focus on feature-based laws? At what point can NF be considered unfair or deceptive to justify regulations by the Federal Trade Commission? Can NF be used for undue influence and how can it be regulated?

The methodology and applications introduced in this special issue continue the excitement and promise offered by NF, including improved therapeutic outcomes without the side effects associated with pharmacological treatments. Key factors include the ethical considerations, importance of individualized approaches, appropriate control conditions, technical limitations and targeted brain regions in NF protocols. However, the critical need for non-pharmacological, non-invasive, precision treatment of a wide range of devastating effects of cognitive and neurological disorders continues to be counterbalanced by the complexity of brain function, physiological heterogeneity, time and expense of NF training. Continued improvements in real-time brain neuroimaging and processing capabilities will enhance the precision and effectiveness of neuromodulation therapies, supporting the development of new therapeutic interventions and expanding the range of treatable conditions.

## Data Availability

This article has no additional data.
